# Single-cell analysis reveals transcriptomic features and therapeutic targets in primary pulmonary lymphoepithelioma-like carcinoma

**DOI:** 10.1038/s42003-025-07819-0

**Published:** 2025-03-08

**Authors:** Binghua Tan, Ke Xu, Yingcheng Lyu, Yicheng Liang, Ruihao Liang, Kai Lei, Jialu Liang, Jing Huang, Kefeng Wang, Duoguang Wu, Wenjian Wang, Xueting Hu, Kexi Wang, Minghui Wang, Huayue Lin

**Affiliations:** 1 Guangdong Provincial Key Laboratory of Malignant Tumor Epigenetics and Gene Regulation, Sun Yat-sen Memorial Hospital, Sun Yat-sen University, Guangzhou, China; 2 Department of Thoracic Surgery, Sun Yat-sen Memorial Hospital, Sun Yat-sen University, Guangzhou, China; 3 Breast Tumor Center, Sun Yat-sen Memorial Hospital, Sun Yat-sen University, Guangzhou, China

**Keywords:** Non-small-cell lung cancer, Molecular medicine, Prognostic markers, Non-small-cell lung cancer

## Abstract

Primary pulmonary lymphoepithelioma-like carcinoma (PPLELC) is a rare subtype of non-small-cell lung cancer. Duo to the current lack of precise targeted therapies, there is an urgent need to identify novel therapeutic targets. In this study, we perform single-nucleus transcriptome analysis on PPLELC samples to reveal the molecular tumor heterogeneity and characterize the functional states of immune cells within the tumor microenvironment. We identify a critical malignant subpopulation of PPLELC characterized by elevated expression of AKT3 and FGFR2. Higher expression levels of AKT3 and FGFR2 are associated with poorer patient outcomes. Moreover, treatment with either an AKT3 inhibitor or an FGFR2 inhibitor significantly attenuates tumor progression in patient-derived xenograft models. Our findings highlight AKT3 and FGFR2 as potential therapeutic targets and prognostic biomarkers, providing valuable insights for the development of rational targeted therapies and immunotherapeutic strategies.

## Abstract (web summary)

Single-cell transcriptome profiling reveals molecular tumor heterogeneity, identifies prognostic markers, and uncovers therapeutic targets for primary pulmonary lymphoepithelioma-like carcinoma, thereby providing clinically relevant insights.

## Introduction

PPLELC is a rare histological type of non-small-cell lung cancer (NSCLC), which is strongly related to Epstein–Barr virus (EBV) infection and accounts for 1% of all cases^1^. With continuous progress in diagnostic techniques, the detection rate of PPLELCs from NSCLC has gradually increased in recent years. PPLELC has unique clinicopathological features, including younger age, non-smoker status and abundant tumor-infiltrating lymphocytes^2^. Frequently mutated driver genes such as epidermal growth factor receptor (EGFR) are rarely detected in PPLELC, which indicates that the patients are less likely to benefit from the current targeted therapy^3^. The first-line treatment strategy for PPLELC is surgery and chemotherapy. Only a small subset of PPLELC patients can benefit from anti-PD-1/ PD-L1 immunotherapy^4^. Nevertheless, post-operative chemotherapy has improved the extremely limited survival time, and immunotherapy has led to hyperprogression for PPLELC patients who are diagnosed in the IIIA phase^5^. This finding highlights the need for personalized therapeutic targets to improve the clinical outcomes of PPLELC patients.

Previously, large-scale genomic studies reveal that the somatic mutation rate is extremely low, but copy number variation (CNV) are widespread in PPLELC according to whole exosome sequencing (WES). However, the clinical application of WES remains less favourable for substantially many PPLELC patients^3,6^. Additionally, the heterogeneity of the tumor microenvironment (TME), resistance to therapies and interpatient discrepancies in clinical responses hinder better prognosis of PPLELC patients. Single-nuclei RNA sequencing (snRNA-seq) offers a unique opportunity to discern diverse arrays of cell types in PPLELC TME. Intercellular interactions foster an immunosuppressive environment to inhibit the eventual prognosis^7^. Understanding the mechanism of these interactions has profound implications for cancer treatment, especially immunotherapies^8^. However, no study has revealed single-cell transcriptional profiles or applied a PDX model to study PPLELC.

AKT3, which is a vital protein kinase B, modulates various cellular activities such as proliferation, survival, apoptosis and tumorigenesis via the PI3K-AKT pathway^9^. The PI3K-AKT pathway was previously reported to be involved in EBV oncogenesis in multiple tumors. Fibroblast growth factor receptor 2 (FGFR2) plays a vital role in lung morphogenesis and contributes to the tumor angiogenesis in most NSCLCs^10^. Inhibitors of AKT3 or FGFR2, which are Enzastaurin or Erdafitinib, have been widely applied in the treatment of several types of cancer as targeted therapies^11–14^. Interestingly, preclinical evidence of the application of Enzastaurin or Erdafitinib in PPLELC patients remains scarce.

Here, we applied snRNA-seq of PPLELC tissues to characterize potential therapeutic targets from a specific malignant subset, the efficacy of which was validated in PDX models. Through single-cell transcriptome profiling, we found a tumor-specific microenvironmental pattern and a functional state of the immune cells. In summary, our work provides a deep insight of the molecular tumor heterogeneity of PPLELC with clinical implications.

## Results

### Single-cell transcriptional profiling in PPLELC

**Fig. 1:**
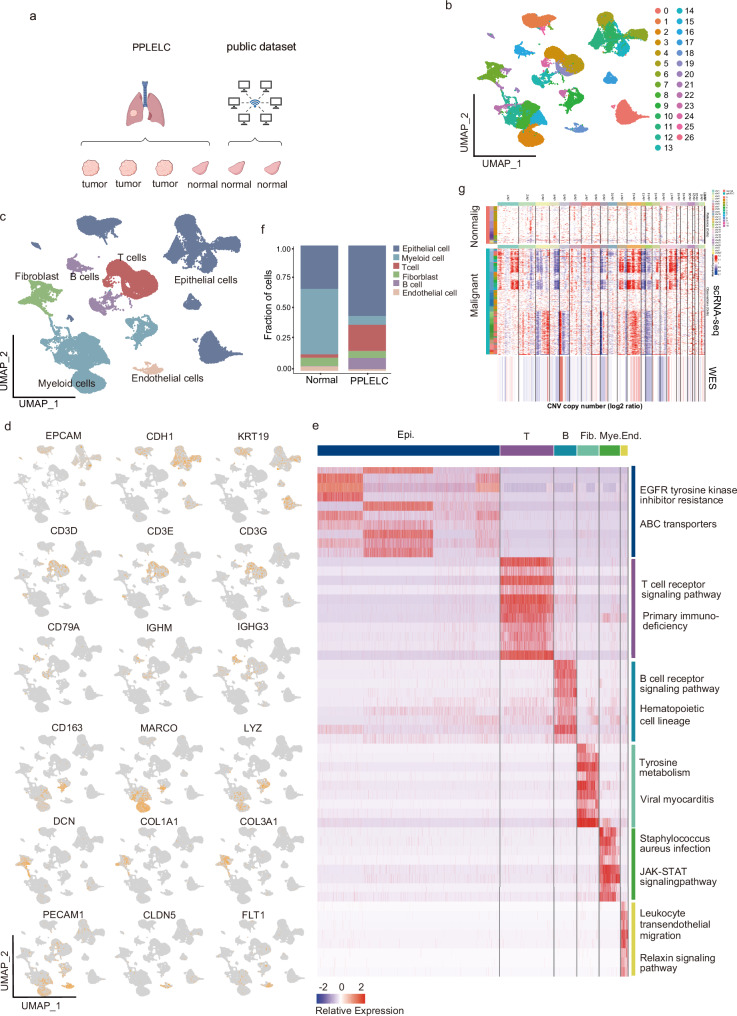
The single-cell transcriptional profiling of human PPLELC. **a** Workflow diagram showing the processing of primary PPLELC tumor and normal tissue for analysis. Created in BioRender. Xu, K. (2025) https://BioRender.com/k27t286. **b** UMAP plots of cells from snRNA-seq with each color representing one cell types. **c** UMAP plot of 50007 single nucleuses grouped into 6 major cell types colored according to cell type and each dot represents one single nucleus. **d** The expression of canonical marker genes for the subsets defined in (**c**). Orange to gray: high to low expression. **e** Heatmap plot showing the marker genes in KEGG Pathway enrichment. **f** The fraction of cell types originating from each sample. Each dot represents one cell type, colored according to sample type. **g** Chromosomal landscape of inferred large-scale CNVs distinguishing malignant epithelial cells from non-malignant epithelial cells. The PPLELC02 tumor is shown with individual cells (y-axis) and chromosomal regions (x-axis). Amplifications (red) or deletions (blue) were inferred by averaging expression over 100-gene stretches on the indicated chromosomes. CNVs are concordant with the calls from WES (bottom). The CNV pattern of the normal epithelial cells from PPLELC04 is also shown (top).

To systematically explore the tumor microenvironment features of PPLELC, we performed snRNA-seq on samples from four independent patients (Fig. 1a, Supplementary Data 1 & 2). After implementing a series of quality controls (Supplementary Data 3) and integrating two publicly available datasets from the GEO database^15^ (GSM4058912 and GSM4058915), 50007 cells were obtained for subsequent analysis, and 6 major cell types were identified in 27 clusters based on the expression of canonical gene markers. The major cell types were epithelial cells, T cells, B cells, myeloid cells, fibroblasts and endothelial cells (Fig. 1b–d, and Supplementary Data 4), and these genes were annotated with the KEGG pathway (Fig. 1e). We observed a significant increase in the proportions of T cells and B cells, whereas the proportion of Myeloid cells was markedly lower in tumors compared to adjacent normal tissues (Fig. 1f). This indicates distinct immune landscapes in PPLELC. Furthermore, although some clinicopathological features and pathogenic factors were shared, the infiltrating immune cells of PPLELC patients were obviously more heterogeneous than those of NPC patients and NSCLC patients, as the previous single-cell publicly available data show (Supplementary Fig. 1a). The malignant cell fraction was greater in PPLELC patients than in NPC patients, whereas the B lymphocyte fraction was greater than that in NSCLC patients. To distinguish malignant cells from non-malignant cells, all epithelial cells were identified based on the identified cell types, and large-scale chromosomal CNVs were inferred in each epithelial cell (Supplementary Fig. 1c)^16^. These CNVs were used to validate the quality of snRNA-seq, which is consistent with the WES data (Fig. 1g).

### A crucial cell cluster recurs across patients and is associated with prognosis in PPLELC patients

**Fig. 2:**
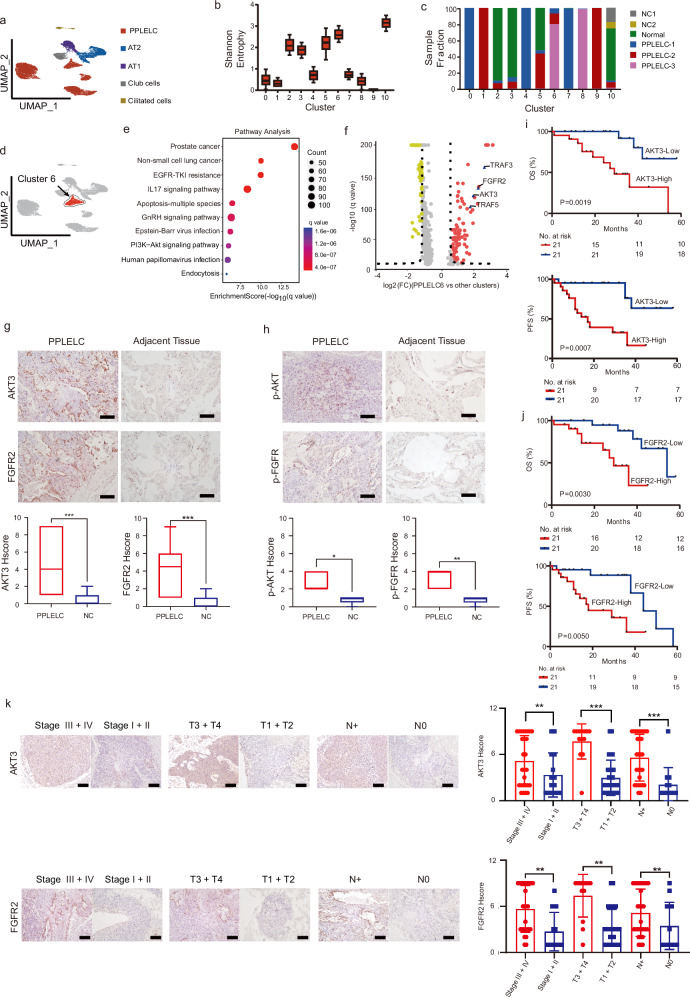
A subpopulation in malignant cell clusters related to prognosis of PPLELC. **a** UMAP plot of 24,127 epithelial cells coloured by different clusters. **b** Boxplot of subtype uncertainty of each SCLC cell stratified by cluster (y axis; measured as entropy of subtype probabilities per cell within each cluster; error bars span the 25th to 75th percentile). **c** Stacked bar plot of sample fraction per cluster as in (**b**). **d** UMAP of PPLELC epithelial cells with recurrent cluster 6 coloured in red. **e** Pathways significantly enriched in cluster 6. Dot plot of ES from GSEA for significantly enriched pathway (*q*-value < 0.05 and ES > 1). **f** Volcano plot of DEGs between cluster 6 and other epithelial cluster identified from snRNA-seq data. **g** Immunohistochemistry assay was performed to detect expression of AKT3 and FGFR2 between tumor and adjacent tissue (top, *n* = 42). Representative images of AKT3 and FGFR2 in PPLELC tumor and adjacent tissue are shown. Scale bars are 200 μm. **h** Immunohistochemistry assay was performed to detect expression of p-AKT and p-FGFR between tumor and adjacent tissue (top, *n* = 42). Representative images of p-AKT and p-FGFR in PPLELC tumor and adjacent tissue are shown. Scale bars are 200 μm. **i**, **j** Overall survival and progression-free progression of 42 PPLELC patients with high or low expression of AKT3 or FGFR2 with the medium Hscore as the threshold. *P*-values are shown in figures. *P*-value of OS was calculated by the log-rank test. *P*-value of FPS was calculated by the multivariate Cox regression test. **k** Immunohistology comparison of expression of AKT3 and FGFR2 in different clinical (left), T (middle) and N (right) stages of PPLELC patients (*n* = 42). Representative images of p-AKT and p-FGFR in different clinical (left), T (middle) and N (right) stages are shown. Scale bars are 200 μm. Data are presented as means ± standard deviation; *P  <  0.05, **P  <  0.01, ***P  <  0.001.

Unsupervised clustering of the PPLELC epithelial cell compartment identified 12 clusters. The epithelial clusters were categorized into 5 subsets based on the expressions of canonical marker genes: PPLELC malignant cells, AT1, AT2, Club cells, basal cells and ciliated cells (Fig. 2a, Supplementary Fig. 1b, and Supplementary Data 4). Most malignant clusters were exclusive to single-tumor tissues, but PPLELC Cluster 6 was strikingly recurrent across all tumor samples but not present in normal samples (Mann-Whitney, *p* < 0.05) (Fig. 2b–d). The cells in PPLELC Cluster 6 had the greatest CNV burden among all epithelial cells, which is consistent with a malignant phenotype (Supplementary Fig. 1c). A heatmap of the epithelial subsets revealed that the DEGs in the epithelial clusters were consistent with the findings of a previous study (Supplementary Fig. 1d).

The genes and gene programs related to viral carcinogenesis and innate immunology, including Epstein-Barr virus infection, IL17signalling pathway and PI3K-AKT signalling pathway, were enriched in the crucial cluster according to the KEGG gene set analysis (Fig. 2e and Supplementary Fig. 2a). A q value of less than 0.05 was used to filter significant changes. The top DEGs between PPLELC Cluster 6 and the other epithelial clusters were analysed to better understand their potential functional differences using a volcano plot (Fig. 2f and Supplementary Data 5). At the gene expression level, TRAF3, AKT3, FGFR2, and TRAF5 were among the most up-regulated genes. The expression levels of both AKT3 and FGFR2 were significantly upregulated in PPLELC samples, as determined by immunohistochemical staining (Fig. 2g and Supplementary Data 6). Consistently, the phosphorylation of AKT and FGFR was also markedly elevated in the PPLELC tissues compared to the control tissues (Fig. 2h and Supplementary Data 6). AKT3 was up-regulated in the Epstein-Barr virus infected cancers progression, such as the proliferation of malignant cells or lymphocytes and immune escape, which might be triggered by EBV-encoded proteins LMP1 and LMP2 in the NPC^17^. FGFR2 pathway is also up-regulated, as implicated in NSCLC, which supports the PPLELC in maintaining or increasing fibroblast proliferation and differentiation^18^.

To investigate the clinical relevance of AKT3 and FGFR2 in PPLELC, we used the median H score as the cut-off value to separate the PPLELC samples into two groups. Notably, Kaplan-Meier survival analysis revealed that the worse overall survival (OS) and progression-free survival (PFS) rates were significantly associated with the increased expression of AKT3 and FGFR2, which supports that patient who highly expressed AKT3 and FGFR2 had a worse prognosis (Fig. 2i, j, Table 1). In addition, the late TNM stage, large tumor size and lymph node metastasis were associated with an increased expression of AKT3 and FGFR2 (Fig. 2k and Supplementary Data 6). Collectively, these results indicate that overexpression of AKT3 and FGFR2 in PPLELC Cluster 6 was positively related to poor prognosis in patients with PPLELC. To explore the specificity of these two biomarkers in PPLELC, we further examined their correlation with overall survival times and TNM stages in LUAD and LUSC. However, no significant associations were observed between AKT3 or FGFR2 expression levels and either the TNM stage or overall survival time in NSCLC patients (Supplementary Fig. 2b & c), highlighting the distinctiveness of PPLELC.

**Table 1:** The relations between clinical characteristic and the expression of AKT3 or FGFR2 in PPLELC patients

	AKT3			FGFR2		
Clinical characteristics	Low	High	*P* value	Low	High	*P* value
Age (y)						
≥ 60	5	10	0.107	4	11	0.024
< 60	16	11		17	10	
Gender						
Male	5	14	0.005	7	12	0.121
Female	16	7		14	9	
Clinical stage						
I	10	0	0.001	9	2	0.031
II	3	1		2	2	
III	8	16		10	13	
IV	1	3		0	4	
T classification						
T1	13	1	0.001	12	2	0.001
T2	7	8		8	7	
T3	0	7		0	7	
T4	1	5		1	5	
N classification						
N0	12	1	0.002	10	3	0.013
N1	2	2		2	2	
N2	5	10		6	9	
N3	2	8		3	7	
Metastasis						
No	21	17	0.115	21	17	0.115
Yes	0	4		0	4	

### Targeted therapies of AKT3 or FGFR2 in PDXs

**Fig. 3:**
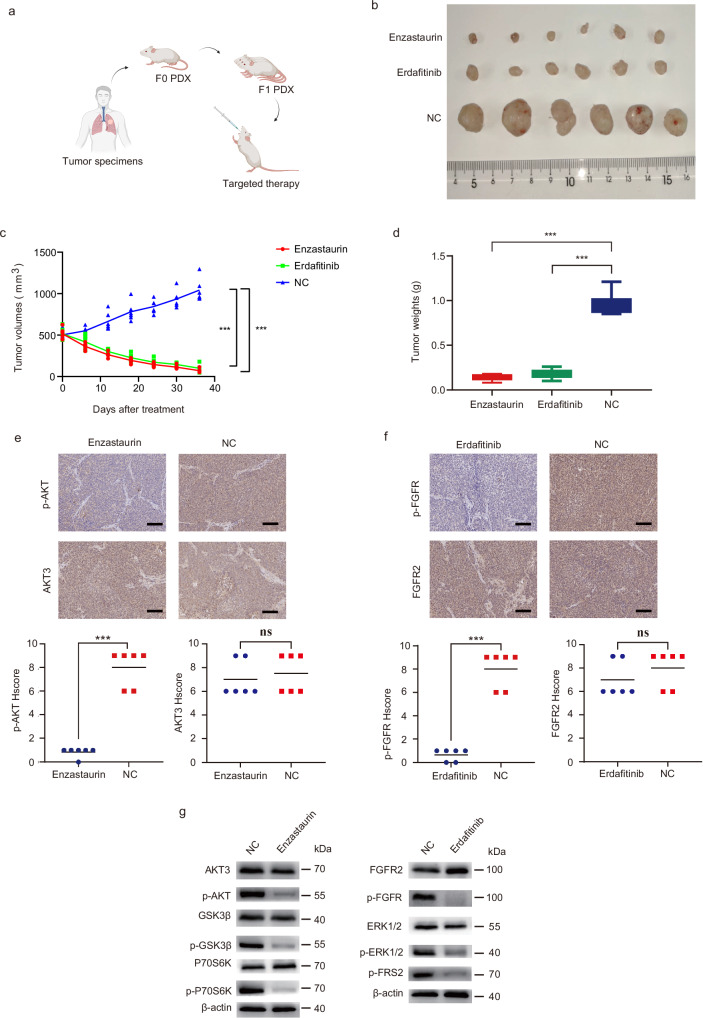
The targeted therapies of AKT3 or FGFR2 shrunk the PPLELC tumor in PDXs. (**a**) Workflow diagram showing the processing of targeting AKT3 and FGFR2 treatments in PPLELC models. Created in BioRender. Xu, K. (2025) https://BioRender.com/p86s709. (**b**) Images of Xenograft tumors in each group of PDXs at the end of experiment (*n* = 6 per group). (**c**)Volume growth curves of xenograft tumor in PDXs (*n* = 6 per group). (**d**) The weight of xenograft tumors in PDXs were measured at the end of the experiment (*n* = 6 per group). *P* value was calculated using unpaired T tests. (**e**) Immunohistologic staining of AKT3 and phosphorylated AKT in xenograft tumors between Enzastaurin and NC groups (*n* = 6 per group) (top). Diagram showing the Hscore results of AKT3 and phosphorylated AKT (*n* = 6 per group) (below). Scale bars are 100 μm. (**f**) Immunohistologic staining of FGFR2 and phosphorylated FGFR in xenograft tumors between Enzastaurin and NC groups (*n* = 6 per group) (top). Diagram showing the Hscore results of FGFR2 and phosphorylated FGFR (*n* = 6 per group) (below). Scale bars are 100 μm. (**g**) The expression of total and phosphorylated AKT, GSK3β and P70S6K between Enzastaurin and NC group (left). The expression of total and phosphorylated of FGFR and ERK1/2 and phosphorylated FRS2 between Erdafitinib and NC group (right). NC, nonsense control. Data are presented as means ± standard deviation; *P  <  0.05, **P  <  0.01, ***P  <  0.001.

To determine whether AKT3 or FGFR2 can serve as potential targets, we first constructed the F0 generation PDX tumor models with biospecimens from PPLELC patients and transplanted into F1 generation PDXs for efficacy experiments. To directly explore the efficiency of the AKT3 inhibitor (Enzastaurin) and FGFR2 inhibitor (Erdafitinib), they were applied to treat PPLELC tumors in vivo (Fig. 3a). The tumor volumes decreased by 93.21% and 90.41% due to Enzastaurin and Erdafitinib compared to the NC group, respectively (*p* < 0.05, ES = 11.90 and 9.13, Fig. 3b, c, and Supplementary Data 7). The tumor weights decreased by 85.26% and 82.11%, whereas no obvious difference was detected in the body weights of the PDX models (*p* < 0.05, ES = 8.30 and 9.13, Fig. 3d, Supplementary Fig. 2d, and Supplementary Data 8 & 9). These results revealed that the tumor growth rate and tumor mass were dramatically inhibited by Enzastaurin or Erdafitinib. To detect the expression of targeted molecules and their downstream proteins, IHC and Western blotting were performed on the tumor tissues. The IHC results showed that the relative phosphorylation over total AKT and relative phosphorylation over total FGFR were significantly suppressed, but the expressions of AKT3 and FGFR2 were unaltered (Fig. 3e, f, and Supplementary Data 10, 11). In addition, the Western blotting results showed that the phosphorylation of downstream proteins including GSK3β, P70S6K, ERK1/2 and p-FRS2 was suppressed compared to the NC group, but there was no significant change in their total expressions (Fig. 3g and Supplementary Fig. 6). Thus, we discovered that both Enzastaurin and Erdafitinib shrank the PPLELC tumor in PDX models, which implies their potential benefit for PPLELC patients.

### T-cell clustering and state analysis in PPLELC

**Fig. 4:**
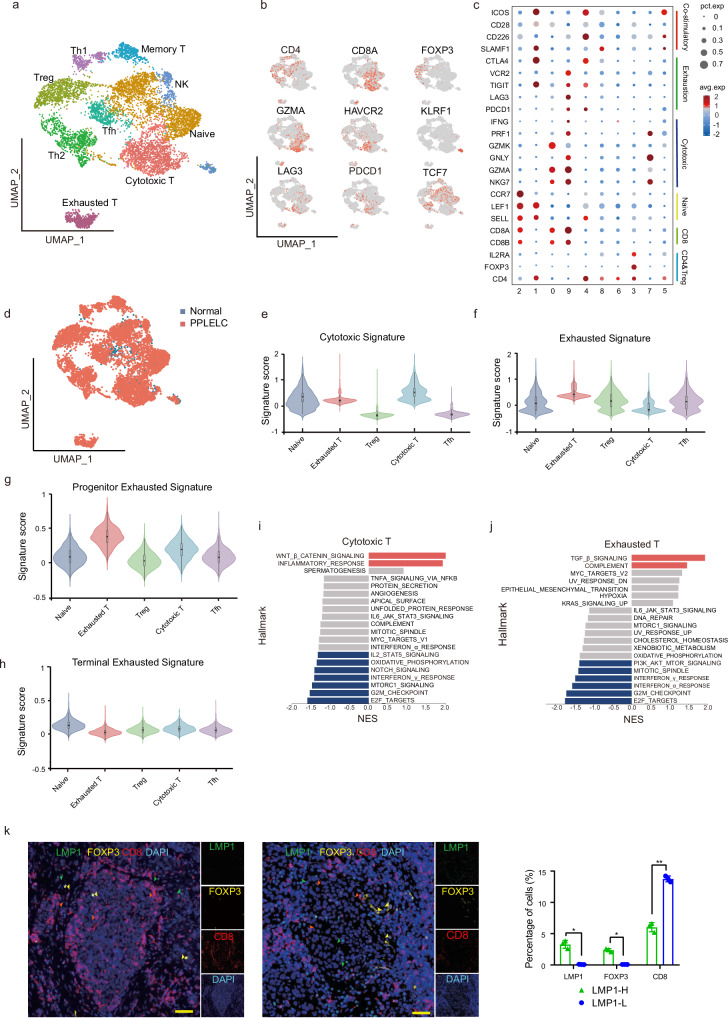
Assessing the functional states of tumor-infiltrating T cells in PPLELC. (**a**) Subclustering of tumor-infiltrating T cells on the UMAP plots of the scRNA-seq datasets. (**b**) Expression and distribution of canonical T cell marker genes among cells. Red to gray: high to low expression. (**c**) Average expression of T cell-specific markers across different clusters. The dot size is proportional to the relative expression level of each gene. (**d**) T cell distribution from tumors and normal tissues. (**e, f**) Violin plots showing the signature scores of cytotoxic and exhaustion gene sets for each tumor-infiltrating T cell cluster in the snRNA-seq. Signature scores for each cell were calculated by the VISION method. (**g, h**) Violin plots showing the signature scores of progenitors and terminal exhaustion gene sets for each tumor-infiltrating T cell cluster in the scRNA-seq. Signature scores for each cell were calculated by the VISION method. (**i, j**) GSEA of significantly enriched pathways for DEGs of cytotoxic (left) and exhausted (right) T cell cluster in the snRNA-seq data. Top significant results ranked by their NES are illustrated. (**k**) Representative images of multiplex IHC staining in PPLELC tumor tissues. Proteins detected using respective antibodies in the assays are indicated on top. The green, yellow, and red arrows indicated the representative cells positive for LMP1, FOXP3 and CD8 proteins in PPLELC tissue, respectively. Scale bars are 200 μm. Data are presented as means ± standard deviation from three independent experiments; *P  <  0.05, **P  <  0.01, ***P  <  0.001.

To better elucidate the heterogeneity of T lymphocyte infiltration in PPLELC, we analysed the gene expression of 7117 T cells from both tumor (6615 cells) and adjacent normal tissues (502 cells). We categorized all T lymphocytes into 10 clusters and annotated these clusters based on their expression of canonical T cell markers and gene molecules among the ten clusters (Fig. 4a). We identified the following clusters: naïve, cytotoxic, exhausted, follicular helper T (Tfh), regulator T (Treg), helper T1 (Th1) and helper T17 (Th17) cells and exhibited their gene profiles (Fig. 4b, c, and Supplementary Data 4). The distribution of T cells was mainly derived from tumors (Fig. 4d). To evaluate the functional state of the major T cell subsets, we removed the clusters from non-malignant tissues with fewer than 150 cells and obtained 5 major T cell subsets, including naïve T cells, exhausted CD8^+^ T cells, Tregs, cytotoxic CD8^+^ T cells and Tfhs.

We next investigated the functional properties of these T cell subsets using multiple functional gene sets and the VISION method^19^ (Supplementary Data 12). The cytotoxic CD8^+^ T cell subsets had the highest cytotoxic score and exhausted the CD8^+^ T cell subsets with the highest exhaustion score and second highest cytotoxic score (*p* < 0.05, Fig. 4e, f), but there were no significant differences in the cell stress signature (Supplementary Fig. 3a). Compared with the other subsets, the exhausted CD8^+^ T cell subset had a greater progenitor exhaustion signature but no obvious difference in the terminal exhaustion signature (Fig. 4g, h). Moreover, pseudo-time analysis was separately applied to CD4^+^ and CD8^+^ T cells to evaluate the conversion. CD4^+^ T cells gradually transformed into Treg and exhausted CD4^+^ T cells (Supplementary Fig. 3b). CD8^+^ T cells exhibited increased cytotoxic and exhausted scores along the trajectory (Supplementary Fig. 3c). Pathway enrichment analysis was performed on the T cell subsets via GSEA (q value < 0.05 and log_2_ (FC) > 1). We found that the T cytotoxicity-related pathways, including the Wnt / β-catenin signalling, inflammatory response and Notch signalling pathways, were significantly enriched in the cytotoxic CD8^+^ T cell subset, whereas Epstein-Barr virus infection might play an important role^20^ (Fig.4i). In addition, the TGF-β catenin pathway, PI3K-AKT pathway and Interferon γ / α pathway^21^ were strongly enriched in the exhausted T cell subset (Fig. 4j), whereas Tregs were strongly positively correlated with the inflammatory response pathway and JAK-STAT3 signalling pathway, which might be induced by lymphocryptoviral latent membrane protein 1 (LMP1), a pathogenic protein encoded by EBV^17,22^ (Supplementary Fig. 3d). Next, paraffin sections from 6 PPLELC patients were subjected to mIHC staining to explore the relationships of LMP1, Treg and CD8^+^ T cells in the PPLELC microenvironment (Fig. 4k and Supplementary Data 13). The results show that the expression of LMP1 protein was significantly positively related to the expression of FOXP3, but negatively related to the expression of CD8 in patient sections.

### B and myeloid cell clustering and state analysis in the PPLELC

**Fig. 5:**
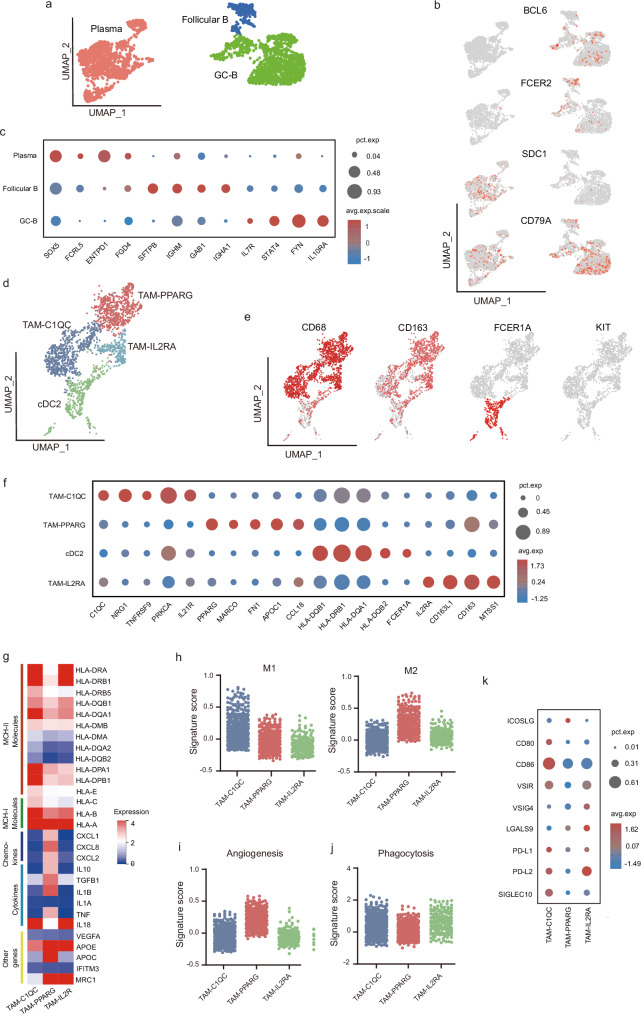
Assessing the functional states of tumor infiltrating-B cells and TAMs in PPLELC. **a** Subclustering of tumor-infiltrating B cells on the UMAP plots of the snRNA-seq datasets. **b** Expression and distribution of canonical B cell marker genes among cells. Red to gray: high to low expression. **c** Dot plot showing the highest expressed genes of each tumor-infiltrating B cell cluster in the snRNA-seq. Dot size indicates the fraction of expressing cells, colored based on normalized expression or activity. **d** Subclustering of TAMs on the UMAP plots of the scRNA-seq datasets. **e** Expression and distribution of canonical TAM marker genes among cells. Red to gray: high to low expression. **f** Dot plot showing the highest expressed genes of each TAM cluster in the snRNA-seq. Dot size indicates the fraction of expressing cells, colored based on normalized expression or activity. **g** Heatmap showing the expression of MHC molecules, chemokines, cytokines, and other related genes in each TAM cluster. **h** Boxplots showing the M1 and M2 signature scores for each TAM cluster in the snRNA-seq data. The signature score was calculated by the VISION method. **i**, **j** Boxplot showing the angiogenesis and phagocytosis signature scores for each TAM cluster in the snRNA-seq data. The signature score was calculated by the VISION method. **k** Dot plot showing the expression of the immune costimulatory, checkpoint, and evasion genes for each TAM cluster in the snRNA-seq dataset.

Furthermore, B lymphocytes support T-cell function and sustain adaptive immunity by generating specific antibodies; thus, they play a prominent role in anti-tumor immunity^23^. We obtained 2907 B cells and divided them into 9 subsets. To accurately explore the B cell clustering and function in PPLELC, we removed the clusters with fewer than 150 cells and applied the canonical B cell markers to define the subsets (Fig. 5a, b, and Supplementary Data 4). Most B lymphocytes were derived from the PPLELC tumor (Fig. 5c and Supplementary Fig. 4a) and defined into three subsets: plasma (SDC1), follicular B cells (FCER2) and germinal centre (GC) B cells (BCL6). GC B cells tend to convert into plasma cells^24^ (Supplementary Fig. 4b). Notably, the plasma subset was significantly down-regulated in the inflammatory pathway (e.g., inflammatory response, Wnt-β and TGF-β pathway, Supplementary Fig. 4c), and IGHM and BACH2 were the highest DEGs. BACH2 has been reported as the immunosuppressive factor in the lung cancer, which indicates poor prognosis^25^.

Tumor-associated macrophages (TAMs) have emerged as a focus in the immune-oncology field because of the vital role of macrophages in tumor progression^26^. Similar to T lymphocyte analysis, we identified three TAM clusters and a cDC2 cluster by applying previously published markers^27^ (Fig. 5d–f, Supplementary Fig. 4d and Supplementary Data 4). The TAM-C1QC cluster highly expressed complement genes (e.g., C1QC, C1QA and C1QB), NRG1 and IL21R. Furthermore, MHC class II molecules, including HLA-DRA, HLA-DRB1 and HLA-DQA1, were highly expressed in the TAM-C1QC cluster, which indicates their vital role in strong antigen presentation ability (Fig. 5g). PPARG^28^ and the lung tissue-resident marker MARCO^27^ were highly expressed in the TAM-PPARG cluster, along with the supersessive genes APOC1^29^, FN1^30^, and CCL18^31^. Moreover, the TAM-PPARG cluster expressed high levels of cytokine and chemokine molecules, which suggests a strong immunosuppressive ability. In the third cluster, TAM-IL2RA highly expressed the innate immune response markers IL2RA^32^ and CD163. MTSS1^33^, which is a novel protein important in tumor progression, was up-regulated in the TAM-IL2RA cluster.

To evaluate the functional state of the TAM subsets in PPLELC, the expression or activity of multiple functional gene sets were detected in their profiles^27^ (Supplementary Data 14). The results revealed that the TAM-C1QC cluster had the highest M1 signature score (*p* < 0.05), followed by the cDC2 cluster (Fig. 5h). Reciprocally, the highest alternative M2 macrophage signature was expressed in the TAM-PPARG cluster compared with the other clusters (*p* < 0.05, Fig. 5h). In addition, the angiogenesis signature and FN1^27^, which are linked with a survival disadvantage in kidney cancer, were more likely associated with the TAM-PPARG cluster, whereas the phagocytosis signature was not significantly different among the three TAM subsets (Fig. 5i, j). In parallel, we investigated the differences among these subpopulations at the pathway level and found that various pathways related to cellular immunity were significantly enriched in the TAM-C1QC cluster (Supplementary Fig. 4e). The TAM-PPARG cluster was enriched in hypoxia and angiogenesis pathways but inversely correlated with inflammation pathways (such as the IFN-γ, IFN-α and JAK-STAT signalling pathways; Supplementary Fig. 4f). Furthermore, we detected the expression of immune checkpoint genes and costimulatory molecules. Multiple costimulatory signals and presentation molecules were detected in the TAM-C1QC cluster but not in the TAM-PPARG or TAM-IL2RA cluster (Fig. 5k). The ligands (VSIR^34^, PD-L1, PD-L2, and SIGLEC10^35^) that mediated the T lymphocyte immune checkpoint were highly expressed in the cluster TAM-C1QC, but ICOSLG^36^ was exclusively enriched in the cluster TAM-PPARG.

### Complex intercellular communication networks in the PPLELC TME

**Fig. 6:**
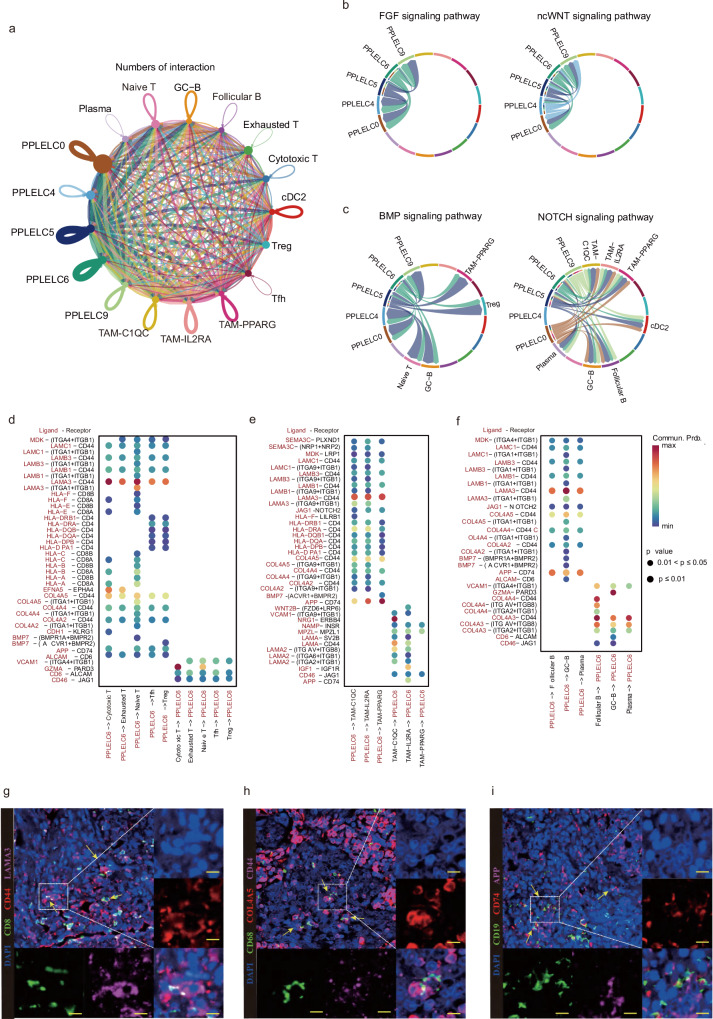
Ligand-receptor-based interaction between tumor and immune cells. **a** Circo plots of the numbers of interaction between cell types in the snRNA-seq data. **b**, **c** Chord plots displaying the pathways enriched in the putative ligand-receptor interactions among malignant cell clusters and other cell types. **d** Dot plots showing the mean interaction strength for selected ligand–receptor pairs between tumor cells and tumor-infiltrating T clusters. Dot size indicates the *p* value, colored by the average expression level of interaction. **e** Dot plots showing the mean interaction strength for selected ligand–receptor pairs between tumor cells and TAM clusters. Dot size indicates the *p* value, colored by the average expression level of interaction. **f** Dot plots showing the mean interaction strength for selected ligand–receptor pairs between tumor cells and tumor-infiltrating B clusters. Dot size indicates the *p* value, colored by the average expression level of interaction. **g** Representative images of mIHC in PPLELC to prove the interaction between tumor cells and T cells. CD8, CD44, LAMA3 and DAPI were labeled with different colors. **h** Representative images of mIHC in PPLELC to prove the interaction between tumor cells and macrophages. CD68, COL4A5, CD44 and DAPI were labelled with different colors. **i** Representative images of mIHC in PPLELC to prove the interaction between tumor cells and B cells. CD19, CD74, APP and DAPI were labelled with different colors.

Cellchat^37^ was used to identify the ligand-receptor pairs and molecular interactions among major cell types (Fig. 6a, Supplementary Fig. 5a, b and Supplementary Data 15). The TAM subsets had the largest number of ligand-receptor interactions, whereas the T subpopulations had the fewest pairs in the immune cell populations. Tumor cells played crucial roles in the entire TME, and the interactions between tumor cells and other immune cells served instructive functions in tumor immunotherapy. The malignant cells were mainly associated with naïve T, Treg, plasma and TAM clusters via multiple ligand-receptor interactions.

In addition, specific signalling pathways regulated by several ligand-receptor pairs between PPLELC and immune cells are shown in the chord plots (Fig. 6b, c, and Supplementary Fig. 5c, d). Notably, the FGF pathway and ncWnt pathway were the major signalling pathways among the malignant PPLELC subsets. Tumor cells that communicated with other immune cells were massively enriched in the BMP, Notch and SEMA3 pathways. To further study the crosstalk between malignant cells and immune cells in the PPLELC TME, we detected major subpopulations of lymphocytes and TAM cells with the greatest number of interactions with malignant PPLELC Cluster 6. Most of the interactions between PPLELC Cluster 6 and T lymphocytes were involved in the ECM-receptor interaction (LAMA3 and CD44)^38^ and cytotoxic function (GZMA and PARD3) (Fig. 6d). In addition, the interactions between malignant PPLELC Cluster 6 and TAM subsets were prominent in terms of macrophage migration inhibitory factor (MIF) and receptors (COL4A5 and CD44), which indicates its vital functions in tumor progression, angiogenesis and immune escape^39^ (Fig. 6e). Unlike the T cell subsets, the three B subpopulations highly expressed CD74 with the ligand APP overexpressed in the tumor cells, which suggests their potential immunosuppressive relation (Fig. 6f). To confirm the reliability of the significantly overexpressed ligand-receptor interactions, multiplex fluorescent immunohistochemical staining was performed on the paraffin sections from PPLELC patients. The results showed that PPLELC tumor cells exhibited notable chemoattraction for CD8^+^ T cells via the crucial interactions of CD44-LAMA3, towards tumor-infiltrating macrophages through COL4A5-CD44, and for B cells through APP-CD74 (Fig. 6g–i). Collectively, our results suggest that the abundant interactions among diverse immune cells and tumor cells may determine the prognosis and reflect therapeutic responses in patients with PPLELC.

## Discussion

In recent years, an increasing number of PPLELC cases have been diagnosed, and there is a lack of effective therapeutic strategies. Our data revealed a unique subset of malignant cells that were recurrent in the tumor tissues of PPLELC patients and presented the highest CNV signals and Shannon entropy, which indicates the distinct characteristics of this malignancy. This malignant subset was characterized by profound expressions of AKT3 and FGFR2. Previous reports have shown that signalling by AKT3 is related to EBV infection in NPC^17^. The vital role of FGFR2 in NSCLC tumorigenesis has been validated by several studies but has not been reported in NPC, so FGFR2 may be an individualized signature for PPLELC. Furthermore, the AKT3 and FGFR2 overexpression is significantly correlated with the late clinical stage, large tumor size, increased lymph node metastasis and poor prognosis. To date, sequencing technology has been applied in the PPLELC with a focus on WES and bulk RNA sequencing^40^. The WES results suggested that mutated genes were rare in PPLELC tissues, which indicates that new therapeutic targets must be urgently explored. In addition, bulk RNA sequencing revealed only down-regulated genes such as ZBTB16, PPARG, TGFBR2 and PIK3R5, in PPLELC patients, and few up-regulated genes were reported. These results indicate the clinical utility of AKT3 or FGFR2 as prognostic markers, but the relationship between AKT3 and FGFR2 in PPLELC and whether EBV infection is the initial factor of the AKT3 and FGFR2 overexpression remain unknown. This AKT3- and FGFR2-overexpressing subcluster constitutes only a small fraction of the malignant cells that comprise PPLELC, but it is strongly associated with survival, which highlights the practical value of single-cell analysis.

Our results revealed that the overexpression of AKT3 and FGFR2 was positively related to poor prognosis in PPLELC patients, which indicates its potential utility as a prognostic biomarker for these patients. However, to further substantiate the clinical application of AKT3 and FGFR2 as biomarkers, strategic validation studies should be conducted in the future. Our study has confirmed the prognostic value of AKT3 and FGFR2 at the pathological level. Future studies will focus on evaluating their clinical validity. To further substantiate the role of these markers, we propose validating the expression levels of AKT3 and FGFR2 in a larger PPLELC cohort, including those from non-research-oriented hospitals. This validation will examine the associations between marker expression and patients’ outcomes (OS, PFS, and EFS), identify appropriate evaluation metrics and diagnostic thresholds, and ultimately establish a standardized evaluation process. Additionally, distinguishing PPLELC from NPC and NSCLC represents a significant challenge for enhancing antitumor therapy. Given the elevated expression levels of AKT3 and FGFR2 in specific subsets of PPLELC cells, which are distinct from NSCLC and NPC, these markers may serve as potential biomarkers for differentiating PPLELC from metastatic NPC and NSCLC, thereby improving clinical outcomes. In future study, we plan to collaborate with other centers to collect specimens of NPC and NSCLC, aiming to further investigate the diagnostic utility of AKT3 and FGFR2.

Currently, surgical resection and chemotherapy are the primary treatments for PPLELC^41^. Resection of the primary tumor is an effective treatment modality for early-stage PPLELC; however, surgery alone is not an independent predictor of OS in these patients. Notably, a previous study reported that traditional staging systems for lung carcinoma may not be appropriate for PPLELC, as the sensitivity to chemotherapy and radiotherapy significantly influences outcomes^42^. Moreover, there is no consensus on the optimal selection of chemotherapy regimens for advanced PPLELC patients, although platinum-based chemotherapy has been identified as an independent predictor for OS. Nevertheless, metastasis and chemoresistance remain significant challenges in PPLELC. Consequently, the development of novel, specific targeted therapeutic drugs is essential. To our knowledge, this study represents the first attempt to establish a PDX model for PPLELC to evaluate therapeutic efficacy. Enzastaurin and Erdafitinib, which have been FDA-approved for clinical use in treating large B-cell lymphoma, colorectal cancer, bladder cancer, urothelial carcinoma and mantle cell lymphoma, demonstrated significant tumor reduction in PPLELC PDX models. Additionally, our sequencing results revealed that CD8^+^ T cells tend to transform into cytotoxic or exhausted T cells, suggesting a potential effective response to immunotherapy. However, while single-agent targeted therapies are generally more effective and less toxic than chemotherapy, they often lead to resistance in advanced NSCLC. A rational combination of targeted therapy, chemotherapy, or immunotherapy may enhance therapeutic efficacy and reduce the development of acquired drug resistance in PPLELC. Taking NSCLC as an example, in the FLAURA2 study^43^, the combination of EGFR-TKI and chemotherapy demonstrated a significant PFS benefit compared to the EGFR-TKI monotherapy group. This suggests that combining targeted therapy with chemotherapy can effectively delay the development of tumor resistance. In future animal model experiments, we intend to establish several groups: monotherapy with targeted agents, combination of targeted therapy and chemotherapy, combination of targeted therapy and immunotherapy, as well as dual-targeted therapy. This will allow us to systematically investigate the optimal utilization of targeted drugs.

Despite promising results in our study, several limitations should be acknowledged. First, due to the challenges associated with preoperative diagnosis and obtaining fresh PPLELC specimens, further cellular mechanistic and functional experiments on tumor cells, as well as additional analyses of immune cells, are warranted. Second, in this study, the patient cohort was exclusively derived from the Chinese population, which may limit the statistical power and generalizability of our findings. For instance, in NSCLC, the mutation frequency of EGFR is markedly higher in Asian populations compared to Caucasian populations, resulting in a greater utilization rate of EGFR-TKIs in Asian NSCLC patients. This ethnic disparity in mutation rates may also be observed in PPLELC. Therefore, future studies should aim to include larger and more diverse populations to validate the correlation between AKT3 and FGFR2 expression levels and prognosis across different ethnicities. Additionally, to balance the sample sizes between tumor and adjacent tissues, we incorporated external single-cell sequencing data of adjacent tissues from GEO datasets. The integration of external datasets may introduce technical and biological biases. To minimize these potential biases, we selected sequencing data generated on the same platform, controlled for confounding factors such as age and gender, and validated key findings in additional samples. In future multi-center clinical studies, we aim to expand the patient cohort size to further reduce the impact of these biases. Third, while AKT3 and FGFR2 show potential as specific prognostic biomarkers for PPLELC patients rather than NSCLC patients, their validity requires confirmation through independent PPLELC cohorts. Finally, prolonged monotherapy with AKT3 or FGFR2 inhibitors may lead to drug resistance, potentially through the activation of alternative signalling pathways and adverse effects on normal cell function. Future studies should explore rational combination therapies that mitigate resistance development and enhance therapeutic efficacy.

In conclusion, our work has revealed that AKT3 and FGFR2 may serve as therapeutic targets and shows the transcriptomic landscape and cell state of the PPLELC microenvironment. Our findings have potential implications for the design of rational targeted therapy and immunotherapeutic approaches.

## Methods

### PPLELC samples

All PPLELC patients were pathologically diagnosed and received on surgery from 2019 to 2022. PPLELC patient were followed-up from 1 to 58 months (median 27.37 months). All tissue specimens from individuals with primary pulmonary lymphoepithelioma-like carcinoma were confirmed by Epstein–Barr virus-encoded small RNA (EBER) staining. We summarized their clinicopathological parameters in Supplementary Data 16. Informed consent was obtained from each patient, and the study protocol was approved by the Ethics Committee of Sun Yat-Sen Memorial Hospital (SYSKY-2024-061-01). All ethical regulations relevant to human research participants were followed. This study is compliant with the Guidance of the Ministry of Science and Technology for the Review and Approval of Human Genetic Resources (2025BAT00067).

### Sample processing, cDNA amplification and library construction

Tween with salts and Tris (TST) buffer were used to isolate PPLELC tissues. PPLELC tissues were mechanically dissociated using fine scissors and nucleus suspension was pelleted by centrifuging at 500 g for 5 min at 4 °C. We added RNase inhibitor (Thermo Fisher Scientific, N8080119) in all TST buffer for all washing steps. Nucleus were resuspended in 200–1000 µl TST buffer, filtered through a 40 µm cell strainer and attached to a fluorescence-activated cell sorting tube (Corning, 352235). The resulting nucleus were counted by disposable counting chambers (Bulldog Bio, DCS-S01), and processed for sequencing. We prepared single-nucleus RNA libraries per Chromium Next GEM Single Cell 3ʹ v3.3 User Guide (10× Genomic) and applied TapeStation D1000 screening tapes (Agilent) and Qubit HS DNA quantification kit (Thermo Fisher Scientific, Q32854) to construct and analyze libraries.

### Processing of snRNA-seq

We generated and processed snRNA-seq data from 10× Genomics platform by CellRanger (version 3.1.0). Firstly, raw base call files were demultiplexed into FASTQ files. Secondly, we performed alignment, quantified the gene expression levels of single nucleus, and align transcripts to the human GRCh38 reference genome. In each sample, cells without gene expression would be removed at the beginning. Next, we applied UMI < 1 and expressed genes < 500 as the filter of low-quality cell. TPM-like value was consequently performed on each cellular gene by the UMI counts divided from the sum of the cellular UMI counts and multiplying by 10,000. Log_2_ (TPM + 1) was set to be the final expression value. Lastly, we applied Seurat package (version 3.2.3) to transform the output file into Seurat object for further analyses.

### Processing of whole-exome sequencing

Whole-Exome Sequencing (WES) from the PPLELC tumor was extracted with Tissue and Blood Kit (Qiagen, 69504). We applied the Agilent SureSelect Human All Exon V6 Kit (Agilent Technologies, 5190-8863) to capture whole-exome and sequenced the resulting libraries by a HiSeq X Ten platform (Illumina). Next, we filtered the sequencing reads with contained adapter reads, low-qualityreads, too many nitrogen atoms (>10%), or low-quality bases (>60% bases with quality < 6). Then we subjected high quality paired-end reads to gap alignment to a UCSC human reference genome (hg19) using BWA-MEM (v0.7.15). Duplicate reads were sorted and marked by Picard (v1.84). Local realignment and base quality score recalibration of the BWA-aligned reads was then conducted using the Genome Analysis Toolkit (GATK; v3.4).

### Inferring CNV based on whole-exome sequencing

PCR duplicates were removed by Samtools rmdup and the coverage depth were calculated at each covered base. The whole exome was segmented into small windows (0.5 Mb in size) and calculated total depth of each window followed by normalization of the sequencing data volume. Using Loess normalization, we corrected the bias from the genomic GC content and calculated depth ratios across the whole genome of each tumor sample. Then we used DNACopy R package to merge windows with similar depth ratios into genomic segments. MergeLevels were further performed to join adjacent segments.

### Integration of individual samples

Batch correction was performed in combining clinical samples reduced to the top 50 PCs using fastMNN with pseudocount of 1. The dataset included three PPLELC samples (PPLELC-1, PPLELC-2, PPLELC-3), one control sample labelled as Normal, and two additional normal samples from the GEO database (GSM4058912 and GSM4058915). An entropy-based measure was performed to evaluate the effect of batch correction, which quantifies the normalized expression mixes across patients^44^. A k-nearest neighbors graph (*k* = 30) was constructed by Euclidean distance on the normalized dataset and computed the fraction of cells qT derived from each tumor sample T in the neighborhood of each cell j. Then Shannon entropy Hj of sample frequencies were calculated within each cell’s neighborhood as:

$${H}_{j}={\sum}_{T}- \, {q}_{T}{{\mathrm{log}}} \, {q}_{T}$$

High entropy suggests that the most similar cells originate from a diverse set of tumors, while low entropy indicates that most similar cells derive from the same tumor. Harmony was run on the PCA matrix above with patient ID as the batch key by default parameters^45^.

### Identification of cell cluster

We identified major cell types using manually annotating of the differentially expressed gene (DEG) between clusters. Cluster-specific markers for each cluster were identified by the “FindAllMarkers” function with fold change threshold as 0.25. The subsets of epithelial, T/NK, B lymphocyte and myeloid cells were identified by canonical markers (Supplementary Data 4). We performed UMAP embeddings to visualize cluster-specific expression scores.

### Differential expressed genes in snRNA-seq and enriched gene pathways

FindAllMarkers were performed on normalized count data to analyze population-specific DEGs. Expression value of each gene in given cluster were compared with other clusters by Wilcoxon rank sum test. Gene set enrichment analysis (GSEA) was performed on DEGs by the GSEA tool (version 4.0.3) based on the Molecular Signatures Database (MSigDB, C5, gene ontology set, version 7.2). The enrichment scores (ES) were calculated based on this ranked DEG list.

### Validation of cancer cells using single-cell CNV calling

Single-cell CNV calling was performed to distinguish cancer cells from non-malignant epithelial compartment. First, all putative cancer subsets were confirmed to separate from the cells originated from normal lung subsets. Additionally, CNVs harboured from cancer cell clusters were identified based on matched bulk DNA-sequencing from our WES data^16^. Meanwhile, we exhibited CNV at the single-cell level with a diploid mean and set available adjacent normal samples as standard deviation. At least two standard deviations from the diploid mean were considered to be a copy number change.

### Analysis of the signatures score in clusters of T lymphocytes and myeloid cells

The expressions of eight signatures (Cytotoxic, Exhausted, Progenitor Exhausted, Terminal Exhausted, M1, M2, Angiogenesis and Phagocytosis, Supplementary Data 12 & 14) were analysed in T lymphocytes and myeloid cells. For each signature, the normalized expression levels of each cellular gene were extracted as a gene expression matrix. Then, for each cell, the average expressed value of all genes in the signature in that cell was regarded as cluster-specific signature score. Finally, a mean overall expression score for the signature was calculated based on all cells of a particular group. Box plots and violin plots were used to compare the signature scores of the cell groups. Wilcoxon-tests were used to analyze the differences in signature scores.

### Cell-cell interaction analysis

We performed Cellchat package to investigate cell–cell interaction among different cell clusters, focusing on those interactions among tumor cells and other immune cells, in PPLELC^37^. To capture interaction, we integrated previously published multiple ligand–receptor resources, resulting in 3190 human ligand–receptor pairs (Supplementary Data 15). We input cell type annotation information, gene-cell raw matrix and these ligand–receptor pairs to cellchat with a threshold set as 0.1. Mean value ≥ 1 and *p*-value < 0.05 were considered as significant ligand–receptor pairs.

### PDX generation of PPLELC and in vivo drug testing

The female NOD-SCID mice aged 5–7 weeks (GemPharmatech, T001492) were randomly divided into 3 groups, housed under pathogen-free conditions, and engrafted with fresh PPLELC tumor fragments measuring 1–8 mm^3^. Enzastaurin (Selleckchem, S1055) was resuspended in 0.05% Tween80, and administered at 100 mg/kg/d for 5 d/week by oral gavage. Erdafitinib (Selleckchem, S8401) was resuspended in 1% Tween 80, and administered at 25 mg/kg/d for 5 d/week by oral gavage. The volume was calculated as (length × width^2^)/2. In animal experiments, the tumor was excised before reaching a maximum volume of 2000 mm³. We ensured that the maximum allowed tumor volume was not exceeded during the experiment. We have complied with all relevant ethical regulations for animal use. Animal studies were approved by the Animal Care and Use Committee of the Guangdong Laboratory Animals Monitoring Institute (IACUC 2023129).

### Immunochemistry

Paraffin-embedded samples were cut into 4 µm consecutive sections, and antigen retrieval by a pressure cooker for 10 min in citric acid buffer (pH 6.0). The FFPE sections were incubated with AKT3 antibody (1:200, Abcam, ab152157), or FGFR2 antibody (1:200, Abcam, ab10648). Hscore were applied to evaluate the protein expression in tumor cells including intensity and percentage of stained tumor cells (Intensity: 0 for no, 1 for weak, 2 for moderate, and 3 for strong; Percentage: 0 for 0%, 1 for 1%–25%, 2 for 26%–50%, 3 for 51%–75%, and 4 for 76%–100%). The Hscore of IHC result was generated as follows: intensity score × percentage score. All PPLELC patients were divided into two subgroups (High and Low) compared to the median Hscore of the AKT3 and FGFR2 immunostaining, respectively. Paraffin-embedded sections of PDXs were obtained after the endpoint of in vivo drug testing. Then, the FFPE samples were incubated with AKT3 antibody (1:200, Abcam, ab152157), FGFR2 antibody (1:200, Abcam, ab10648), p-AKT antibody (1:100, Cell Signaling Technology, 4060) and p-FGFR antibody (1:50, Thermo Fisher, PA5-105880).

### Multi-IHC

FFPE slides from 6 PPLELC primary tumors from a cohort of 42 PPLELC patients including snRNA-seq samples, were subjected to multi-IHC and multispectral imaging using a PANO Multiplex IHC kit (Panovue, 10004100100) to examine LMP1 (1:100, Santa Cruz, sc-71023), CD8 (1:200, Abcam, ab237709), FOXP3 (1:200, Abcam, ab20034), CD19 (1:500, Abcam, ab134114), CD68 (1:1000, Abcam, ab303565), LAMA3 (1:200, Abcam, ab151715), CD44 (1:50, Cell Signaling Technology, 3570), CD74 (1:200, Cell Signaling Technology, 77274), APP (1:500, Cell Signaling Technology, 29765 T) and COL4A5 (1:200, Abcam, ab157779). The whole steps of mIHC were followed the manufacture’s protocol^46^.

### Western blotting

Western blotting was performed as described in ref. ^43^ using AKT3 (1:5000, Abcam, ab152157), FGFR2 (1:2000, Abcam, ab10648), p-AKT (1:1000, Cell Signaling Technology, 4060), p-FGFR (1:1000, Thermo Fisher, PA5-105880), GSK3β (1:5000, Abcam, ab32391), p-GSK3β (1:1000, Thermo Fisher, 44-604 G), P70S6K (1:1000, Cell Signaling Technology, 2708), p-P70S6K (1:1000, Thermo Fisher, PA5-104842), ERK1/2 (1:1000, Thermo Fisher, 13-6200), p-ERK1/2 (1:1000, Thermo Fisher, 44-680 G), p-FRS2 (1:1000, Thermo Fisher, PA5-118578) and β-Actin (1:1000, Cell Signaling Technology, 4967).

### Statistics and reproducibility

Statistical analysis was performed and plots were generated using GraphPad Prism 9 and SPSS Statistics 26. Q value Correction were applied to correct the calculated *p* value in differential expressed genes, and q value < 0.05 was considered significant. *P*-value ≥ 0.05 were written as ns., *p*-value < 0.05, < 0.01, and < 0.001 are represented as *, **, and ***, respectively. The overall survival analyses were performed using the Log-rank test, and *p* < 0.05 was considered significant. The *p* value of progression-free survival was calculated using the multivariate Cox regression test, and *p* < 0.05 was considered significant. The effect size in PDX models was calculated by Cohen’s d formula. To ensure reproducibility, four PPLELC tumor samples for snRNA-seq and 42 PPLELC patients’ tumor samples served as biological replicates. Other quantification methods and analysis had been described for each method.

### Reporting summary

Further information on research design is available in the Nature Portfolio Reporting Summary linked to this article.

## Supplementary information

Supplementary Information

Supplementary Data 1–16

Description of Additional Supplementary Materials

Reporting Summary

## Data Availability

The raw sequence data reported in this paper have been deposited in the Genome Sequence Archive in National Genomics Data Center, China National Center for Bioinformation / Beijing Institute of Genomics, Chinese Academy of Sciences (GSA-Human: HRA009335) that are publicly accessible at https://ngdc.cncb.ac.cn/gsa-human^47^. The raw data for all the figures can be found in Supplementary Data. Uncropped Western blot images are available in the Supplementary Information. The datasets used and/or analyzed during the current study are available from the corresponding author upon reasonable request.
