# Supplementary material for: Single-cell analysis reveals transcriptomic features and therapeutic targets in primary pulmonary lymphoepithelioma-like carcinoma
Source: Commun Biol. 2025 Mar 8;8:394. doi: 10.1038/s42003-025-07819-0 (PMC11890618; doi:10.1038/s42003-025-07819-0)
Supplement: Supplementary file 1 — Supplementary Information [file 42003_2025_7819_MOESM1_ESM.pdf]

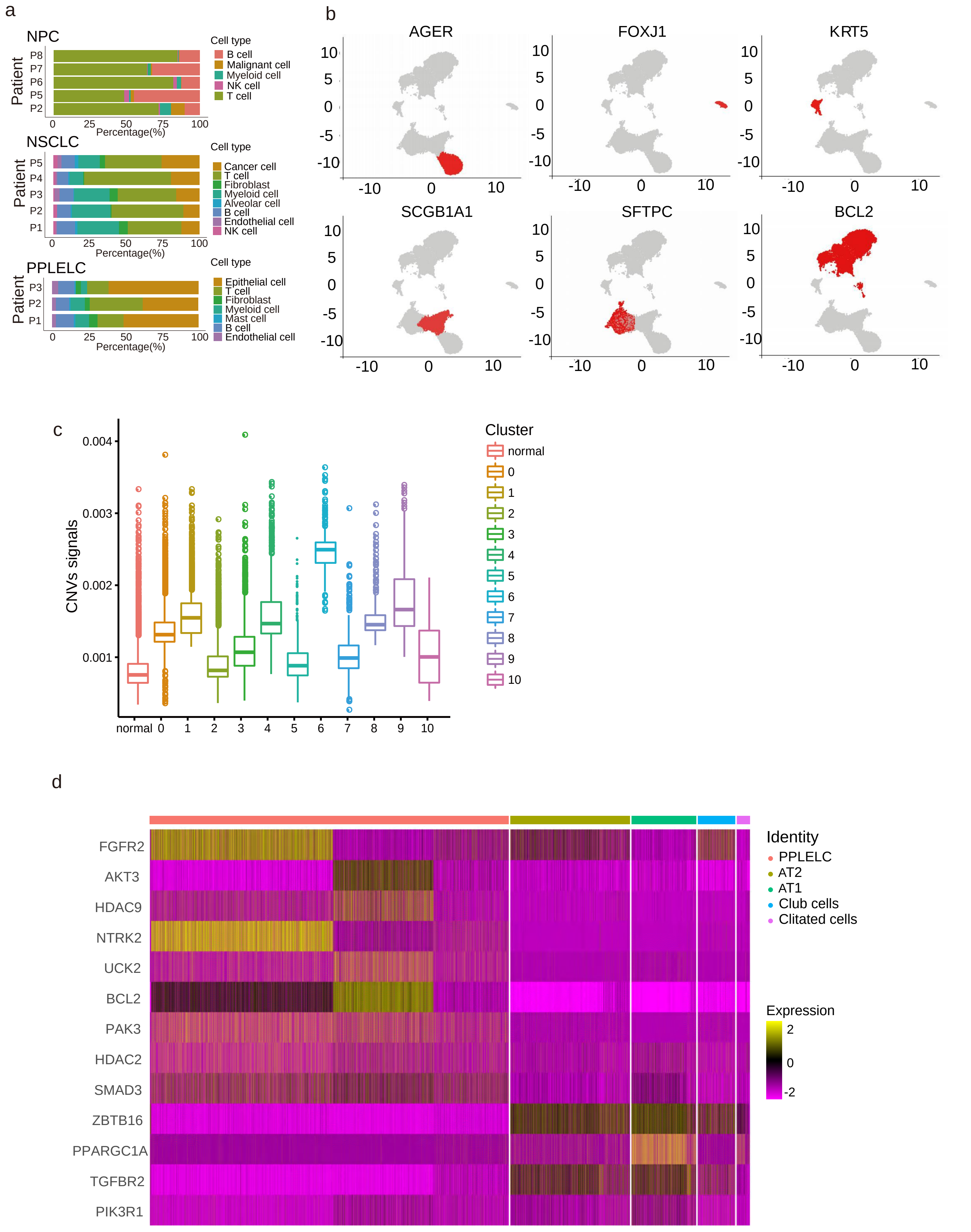

Supplementary Figure 1. Additional information of epithelial cell subsets in PPLELC. a, different cell type fractions in NPC, NSCLC and PPLELC. b, Expression and distribution of canonical T cell marker genes among cellsc, CNV signals of every epithelial subset in PPLELC. d, Heatmap of expression of every epithelial subset in PPLELC.

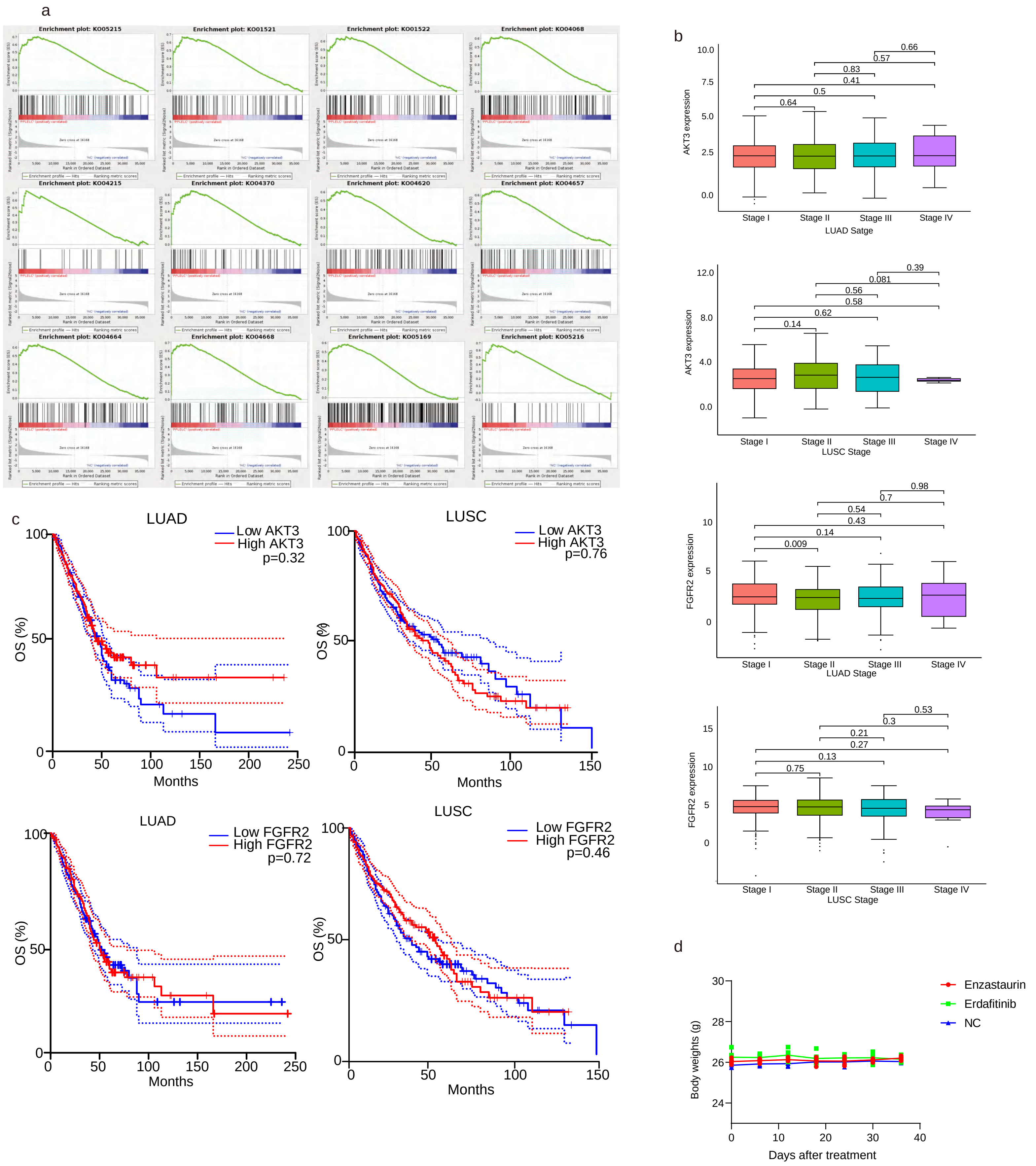

Supplementary Figure 2. Additional information of PPLELC 6. a, Enriched pathway of DEGs in PPLELC 6 by GSEA. b, Box plot showed the relationships between AKT3 or FGFR2 and clinical stages of LUAD and LUSC. c, Overall survival of LUAD (n=478) and LUSC patients(n=484) with high or low expression of AKT3 or FGFR2 with the medium expression as the threshold. P-values are shown in figures. d, Body weights curves of PDXs(n=6 per group, unit:mg).

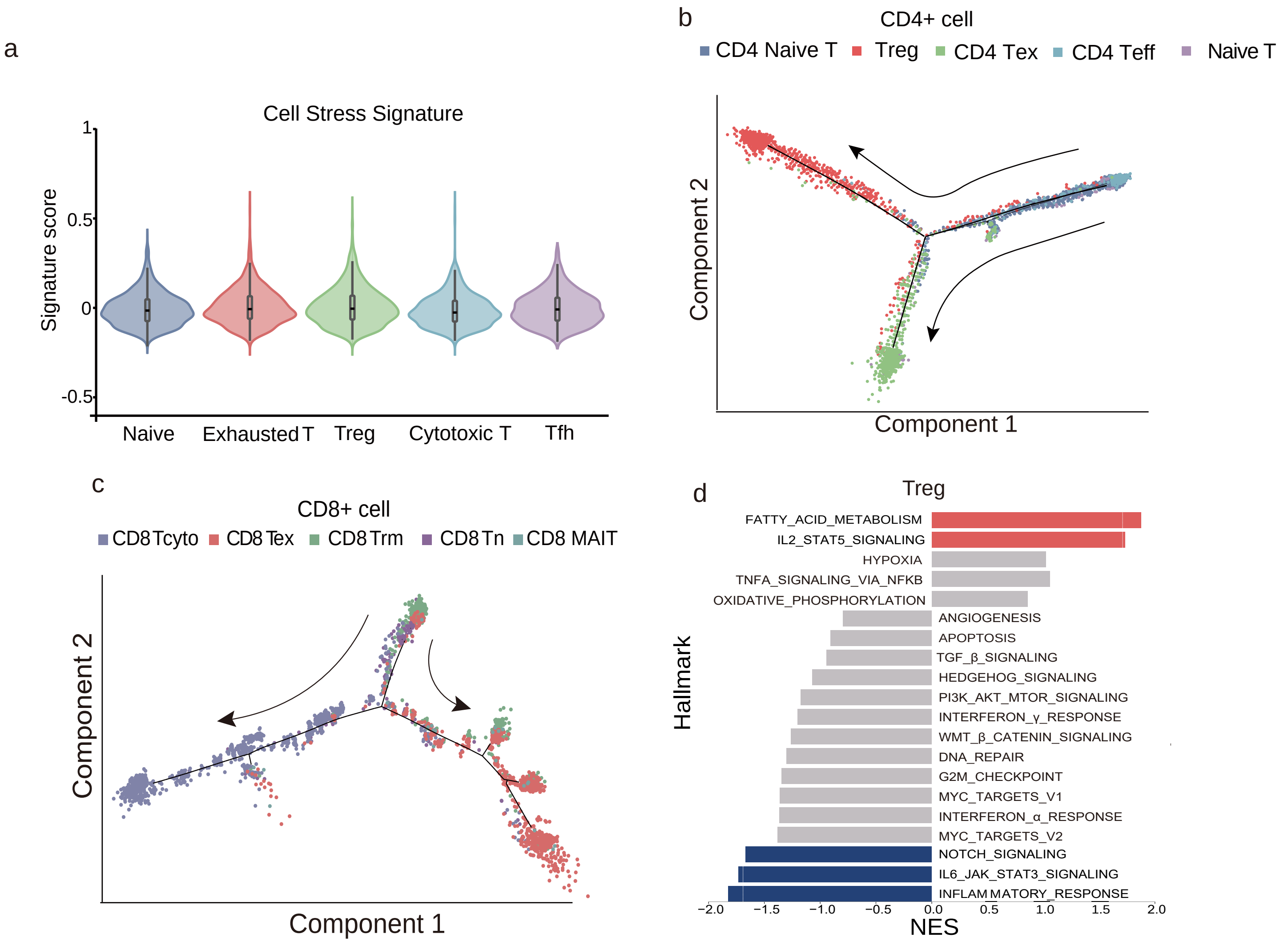

Supplementary Figure 3. Additional information of tumor-infiltrating T cells in PPLELC. a, Violin plots showing the signature scores of cell stress gene sets for each tumor-infiltrating T cell cluster. b, The developmental trajectory of CD4+ T cells inferred by Monocle2. c, The developmental trajectory of CD8+T cells inferred by Monocle2. d, GSEA of significantly enriched pathways for DEGs of Treg.

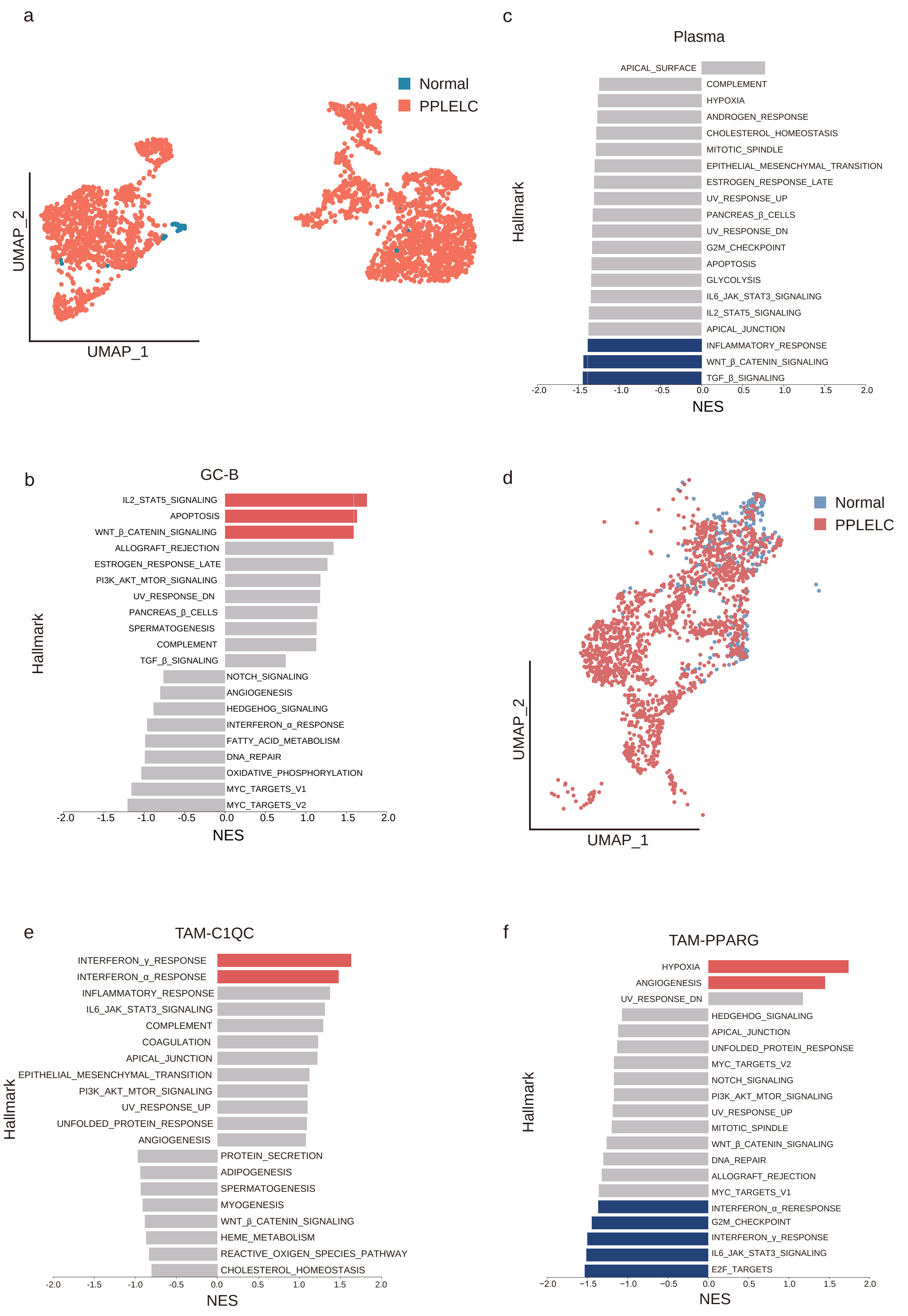

Supplementary Figure 4. Additional information of B cells and macrophages in PPLELC. a, B cell distribution from tumors and nonmalignant tissues. b, GSEA of significantly enriched pathways for DEGs of GC-B. c, GSEA of significantly enriched pathways for DEGs of plasma. d, Macrophage distribution from tumors and nonmalignant tissues. e, GSEA of significantly enriched pathways for DEGs of TAM-C1QC. f, GSEA of significantly enriched pathways for DEGs of TAM-PPARG.

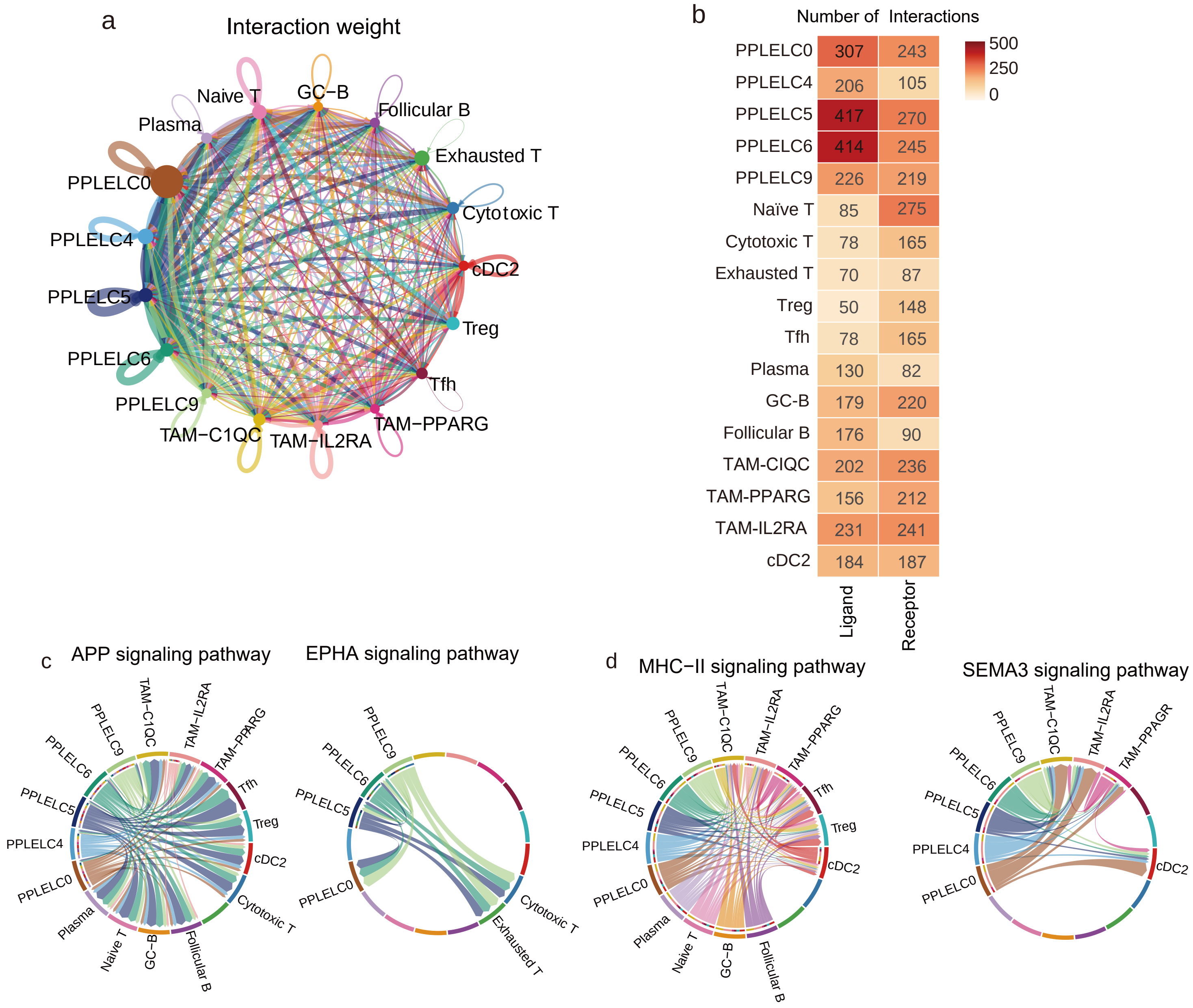

Supplementary Figure 5. Additional information of cell interactions in PPLELC. a, Circo plots of the weights of interaction between cell types in the snRNA-seq data. b, the details of the number of interaction btween cell types. c, d, Chord plots displaying the pathways enriched in the putative ligand-receptor interactions between malignant cell clusters and other cell types.

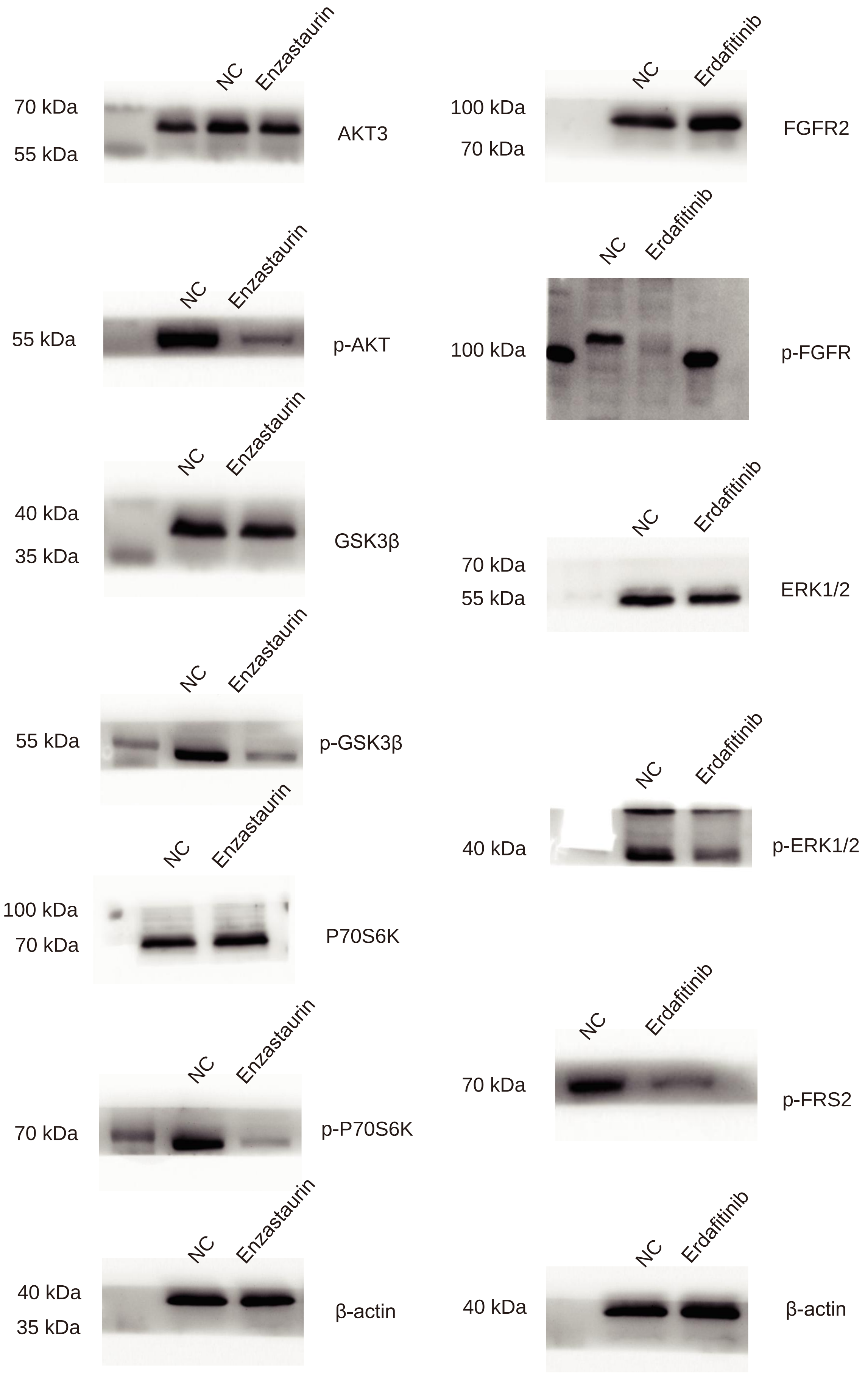

Supplementary Figure 6. Unprocessed scans of western blots presented in Figure 3g.
