# Supplementary material for: Single-cell analysis reveals transcriptomic features and therapeutic targets in primary pulmonary lymphoepithelioma-like carcinoma
Source: Commun Biol. 2025 Mar 8;8:394. doi: 10.1038/s42003-025-07819-0 (PMC11890618; doi:10.1038/s42003-025-07819-0)
Supplement: Supplementary file 3 — Description of Additional Supplementary Materials [file 42003_2025_7819_MOESM3_ESM.pdf]

Description of Additional Supplementary Files

File name: Supplementary Data 1

Description: Clinicopathological Information of Samples for Single-nucleus RNA-seq

File name: Supplementary Data 2

Description: Cell fractions of 3'droplet-based single-nucleus RNA-seq data

File name: Supplementary Data 3

Description: Information of 3'droplet-based single-nucleus RNA-seq data.

File name: Supplementary Data 4

Description: List of canonical markers to assign the cell types and their references.

File name: Supplementary Data 5

Description: Top 50 differential expressed genes of PPLELC 6 subsets by snRNA-seq(tumor vs. nonmalignant tissue)

File name: Supplementary Data 6

Description: Source data of Figure 2g, 2h and 2k

File name: Supplementary Data 7

Description: Source data of Figure 3c

File name: Supplementary Data 8

Description: Source data of Figure 3d

File name: Supplementary Data 9

Description: Source data of supplementary Figure 2d

File name: Supplementary Data 10

Description: Source data of Figure 3e

File name: Supplementary Data 11

Description: Source data of Figure 3f

File name: Supplementary Data 12

Description: Functional state gene sets T cell

File name: Supplementary Data 13

Description: Source data of Figure 4k

File name: Supplementary Data 14

Description: Functional state genes of macrophages

File name: Supplementary Data 15

Description: List of the interactions of liangd-receptor

File name: Supplementary Data 16

Description: Baseline Characteristics of PPLELC patients
