# Supplementary material for: Single-cell analysis reveals transcriptomic features and therapeutic targets in primary pulmonary lymphoepithelioma-like carcinoma
Source: Commun Biol. 2025 Mar 8;8:394. doi: 10.1038/s42003-025-07819-0 (PMC11890618; doi:10.1038/s42003-025-07819-0)
Supplement: Supplementary file 4 — Reporting Summary [file 42003_2025_7819_MOESM4_ESM.pdf]

nature portfolio

Corresponding author(s): Minghui Wang

Last updated by author(s): Feb 11, 2025

Reporting Summary

Nature Portfolio wishes to improve the reproducibility of the work that we publish. This form provides structure for consistency and transparency in reporting. For further information on Nature Portfolio policies, see our Editorial Policies and the Editorial Policy Checklist.

Statistics

For all statistical analyses, confirm that the following items are present in the figure legend, table legend, main text, or Methods section.

n/a	Confirmed	
☐	☒	The exact sample size (n) for each experimental group/condition, given as a discrete number and unit of measurement
☐	☒	A statement on whether measurements were taken from distinct samples or whether the same sample was measured repeatedly
☐	☒	The statistical test(s) used AND whether they are one- or two-sided<br>*Only common tests should be described solely by name; describe more complex techniques in the Methods section.*
☐	☒	A description of all covariates tested
☐	☒	A description of any assumptions or corrections, such as tests of normality and adjustment for multiple comparisons
☐	☒	A full description of the statistical parameters including central tendency (e.g. means) or other basic estimates (e.g. regression coefficient) AND variation (e.g. standard deviation) or associated estimates of uncertainty (e.g. confidence intervals)
☐	☒	For null hypothesis testing, the test statistic (e.g. F, t, r) with confidence intervals, effect sizes, degrees of freedom and P value noted<br>*Give P values as exact values whenever suitable.*
☒	☐	For Bayesian analysis, information on the choice of priors and Markov chain Monte Carlo settings
☒	☐	For hierarchical and complex designs, identification of the appropriate level for tests and full reporting of outcomes
☐	☒	Estimates of effect sizes (e.g. Cohen's d, Pearson's r), indicating how they were calculated

Our web collection on statistics for biologists contains articles on many of the points above.

Software and code

Policy information about availability of computer code

Data collection	Our snRNA-seq data were collected by Chromium Next GEM Single Cell 3′ v3.3 reagents(10X Genomics). The published scRNA-seq data of human Lung, NSCLC and NPC used in this study are available in the GEO database under accession code GSE4058912, GSE4058915, GSE99254 and GSE162025.
Data analysis	In this study,we used software general workflow codes,without generating any new code. The R Project for Statistical Computing:R(v4.0.2); Pre-processing and quality control of snRNA-seq : Cell Ranger (v3.1.0);Integration of snRNA-seq datasets:Seurat (v 3.2.3); Pseudotime trajectory analysis:Monocle 2;Gene ontology (GO)enrichment analysis:KOBAS 3.0; Calculate signature score: VISION (v 2.0.0); Gene Set Enrichment Analysis(GSEA) analysis: GSEA (v 4.0.3); Cell - cell interaction:CellChat (https://github.com/sqjin/CellChat); inferCNV(https://github.com/broadinstitute/inferCNV); Calculate H-score in IHC staining: ImageJ (150).

For manuscripts utilizing custom algorithms or software that are central to the research but not yet described in published literature, software must be made available to editors and reviewers. We strongly encourage code deposition in a community repository (e.g. GitHub). See the Nature Portfolio guidelines for submitting code & software for further information.

Data

Policy information about availability of data

All manuscripts must include a data availability statement. This statement should provide the following information, where applicable:

- Accession codes, unique identifiers, or web links for publicly available datasets
- A description of any restrictions on data availability
- For clinical datasets or third party data, please ensure that the statement adheres to our policy

Source data of single nucleus RNA sequencing has been submitted to GSA Human database and is available from the corresponding authors on reasonable request. Other data are available from the corresponding authors on reasonable request.

Human research participants

Policy information about studies involving human research participants and Sex and Gender in Research.

Reporting on sex and gender	The participant includes 23 female and 19 male.
Population characteristics	35.7% participants are over 60 years old and 64.3% are under 60 years old. 81.0% participants are non-smokers and 19.0% are smokers or have a history of smoking.
Recruitment	42 patients with primary pulmonary lymphoepithelioma-like carcinoma are confirmed by EBER staining after surgery.
Ethics oversight	The study protocol was approved by the Ethics Committee of Sun Yat-Sen Memorial Hospital (SYSKY-2024-061-01).

Note that full information on the approval of the study protocol must also be provided in the manuscript.

Field-specific reporting

Please select the one below that is the best fit for your research. If you are not sure, read the appropriate sections before making your selection.

☒ Life sciences ☐ Behavioural & social sciences ☐ Ecological, evolutionary & environmental sciences

For a reference copy of the document with all sections, see nature.com/documents/nr-reporting-summary-flat.pdf

Life sciences study design

All studies must disclose on these points even when the disclosure is negative.

Sample size	We include 42 patients diagnosed as PPLELC in this study and 18 NOD. mice were used to establish PDX models.
Data exclusions	Filters were applied to keep nuclei with 200–7,500 genes, 400–40,000 unique molecular identifiers (UMIs), and less than 10% mitochondrial reads. In addition, Scrublet was applied to identify and remove doublets with an expected doublet rate ranging from 4% to 9.6% based on the loading rate.
Replication	This study had no technical replicates.
Randomization	All the mice were were randomly divided into 3 groups.
Blinding	The investigator was blinded to the group allocation when assessing the outcome.

Reporting for specific materials, systems and methods

We require information from authors about some types of materials, experimental systems and methods used in many studies. Here, indicate whether each material, system or method listed is relevant to your study. If you are not sure if a list item applies to your research, read the appropriate section before selecting a response.

Materials & experimental systems

n/a	Involved in the study
☐	☒ Antibodies
☒	☐ Eukaryotic cell lines
☒	☐ Palaeontology and archaeology
☐	☒ Animals and other organisms
☒	☐ Clinical data
☒	☐ Dual use research of concern

Methods

n/a	Involved in the study
☒	☐ ChIP-seq
☒	☐ Flow cytometry
☒	☐ MRI-based neuroimaging

Antibodies

Antibodies used

Antibody Catalogue # (Company)
AKT3 ab152157(abcam)
FGFR2 ab10648(abcam)
p-AKT #4060(Cell Signaling Technology)
p-FGFR PA5-105880(ThermoFisher)
GSK3β ab32391(abcam)
p-GSK3β 44-604G(ThermoFisher)
P70S6K #2708(Cell Signaling Technology)
p-P70S6K PA5-104842(ThermoFisher)
ERK1/2 13-6200(ThermoFisher)
p-ERK1/2 44-680G(ThermoFisher)
p-FRS2 PA5-118578(ThermoFisher)
β-Actin #4967(Cell Signaling Technology)
LMP1 sc-71023(Santa Cruz Biotechnology)
CD8 ab237709(abcam)
FOXP3 ab20034(abcam)
p-AKT #4060(Cell Signaling Technology)
p-FGFR PA5-105880(ThermoFisher)
CD19 ab134114(abcam)
CD68 ab303565(abcam)
LAMA3 ab151715(abcam)
CD44 #3570(Cell Signaling Technology)
CD74 #77274(Cell Signaling Technology)
APP #29765T(Cell Signaling Technology)
COL4A5 # ab157779(abcam)
DAPI 62248(ThermoFisher)

Validation

Validation statements are available from manufacturers' websites:
AKT3 https://www.abcam.cn/products/primary-antibodies/akt3-antibody-ab152157.html
FGFR2 https://www.abcam.cn/products/primary-antibodies/fgfr2-antibody-ab10648.html
p-AKT https://www.cellsignal.com/products/primary-antibodies/phospho-akt-ser473-d9e-xp-rabbit-mab/4060
p-FGFR https://www.thermofisher.cn/cn/zh/antibody/product/Phospho-FGFR2-Tyr769-Antibody-Polyclonal/PA5-105880
GSK3β https://www.abcam.cn/products/primary-antibodies/gsk3-beta-antibody-y174-ab32391.html
p-GSK3β https://www.thermofisher.cn/cn/zh/antibody/product/Phospho-GSK3B-Tyr216-Tyr279-Antibody-Polyclonal/44-604G
P70S6K https://www.cellsignal.cn/products/primary-antibodies/p70-s6-kinase-49d7-rabbit-mab/2708
p-P70S6K https://www.thermofisher.cn/cn/zh/antibody/product/Phospho-p70-S6-Kinase-Thr389-Thr412-Antibody-Polyclonal/PA5-104842
ERK1/2 https://www.thermofisher.cn/cn/zh/antibody/product/ERK1-ERK2-Antibody-clone-ERK-7D8-Monoclonal/13-6200
p-ERK1/2 https://www.thermofisher.cn/cn/zh/antibody/product/Phospho-ERK1-ERK2-Thr185-Tyr187-Antibody-Polyclonal/44-680G
p-FRS2 https://www.thermofisher.cn/cn/zh/antibody/product/Phospho-FRS2-Tyr436-Antibody-Polyclonal/PA5-118578
β-Actin https://www.cellsignal.cn/products/primary-antibodies/b-actin-antibody/4967
LMP1 https://www.scbt.com/zh/p/ebv-lmp-1-antibody-3h2104-a-b-c
CD8 https://www.abcam.cn/products/primary-antibodies/cd8-alpha-antibody-cal66-ab237709.html
FOXP3 https://www.abcam.cn/products/primary-antibodies/foxp3-antibody-236ae7-ab20034.html
CD19 https://www.abcam.cn/products/primary-antibodies/cd19-antibody-epr5906-ab134114.html
CD68 https://www.abcam.cn/products/primary-antibodies/cd68-antibody-rm1031-ab303565.html
LAMA3 https://www.abcam.cn/products/primary-antibodies/lama3-antibody-epr8266-ab151715.html
CD44 https://www.cellsignal.com/products/primary-antibodies/cd44-156-3c11-mouse-mab/3570
CD74 https://www.cellsignal.cn/products/primary-antibodies/cd74-d5n3i-xp-rabbit-mab/77274
APP https://www.abcam.cn/products/primary-antibodies/col4a5-antibody-ab157779.html
COL4A5 https://www.abcam.cn/products/primary-antibodies/col4a5-antibody-ab157779.html
DAPI https://www.thermofisher.cn/order/catalog/product/62248

Animals and other research organisms

Policy information about studies involving animals; ARRIVE guidelines recommended for reporting animal research, and Sex and Gender in Research

Laboratory animals	NOD. mice age 5–7-week were used.
Wild animals	The study did not involve wild animals.
Reporting on sex	All the mice were female.
Field-collected samples	The study did not involve samples collected from the field.
Ethics oversight	All the experiments were performed following the experimental protocol approved by the Animal Ethics Committee(IACUC 2023129).

Note that full information on the approval of the study protocol must also be provided in the manuscript.
